# Spontaneous Proteus mirabilis Meningitis in Adults Requiring an Extended Antibiotic Course: Case Report and Literature Review

**DOI:** 10.7759/cureus.39225

**Published:** 2023-05-19

**Authors:** Francisco F Costa Filho, Alan Furlan, Benjamin S Avner

**Affiliations:** 1 Internal Medicine, Western Michigan University Homer Stryker M.D. School of Medicine, Kalamazoo, USA

**Keywords:** gram-negative bacillary meningitis, adult bacterial meningitis, atypical meningitis, bacterial meningitis, proteus mirabilis

## Abstract

Most cases of gram-negative bacillary meningitis occur in neonates and infants. Meningitis in adults caused by *Proteus mirabilis* has been reported rarely. Evidence-based guidelines for the treatment of adult patients with gram-negative bacillus meningitis are scarce. The optimal duration of antibiotic therapy for these patients is an unanswered question in the medical literature. This article outlines a case of community-acquired meningitis caused by *P. mirabilis* in an adult patient who required an extended antimicrobial treatment, after failing to a three-week antibiotic regime.

Our patient, a 66-year-old man with a history of neurogenic bladder, remote spinal cord trauma, and recurrent urinary tract infections presented to the emergency department reporting a two-day history of severe headache, fever, and confusion. Cerebrospinal fluid (CSF) revealed significant neutrophil-predominant pleocytosis, low glucose level, and elevated protein level. CSF culture grew few pan-susceptible *P. mirabilis*. The patient initially completed 21 days of ceftriaxone guided by susceptibility testing. Nine days after finishing antibiotic therapy, the patient was readmitted with recurrent headache, fever, and neck rigidity. A new CSF study again revealed pleocytosis, elevated polymorphonuclear cells, low glucose level, and elevated protein level, but with a negative CSF culture. The patient became afebrile, and his symptoms improved after two days of ceftriaxone. He completed an additional six-week regime of ceftriaxone. On the one-month follow-up visit, the patient remained afebrile, with no recurrent symptoms.

Spontaneous community-acquired *P. mirabilis* meningitis is rare among adult patients. Experiences in the treatment of gram-negative bacillus meningitis in adults must be shared with the scientific community to build up a better understanding of this condition. In the context of this case, sterilization of the CSF, extended antibiotic therapy, and a close post-treatment follow-up are crucial for treating this life-threatening condition.

## Introduction

Bacterial meningitis is a medical emergency and should be recognized and treated promptly. There are well-established guidelines in the medical literature for the management of bacterial meningitis caused by common pathogens such as *Streptococcus pneumoniae*, *Neisseria meningitidis*, *Haemophilus influenzae*, *Listeria monocytogenes*, and group B streptococci [1]. Due to its infrequency, however, evidence-based guidelines for the treatment of adult patients with gram-negative bacillus meningitis are scarce. Most cases of gram-negative meningitis occur in neonates or infants [2]. Adult cases of gram-negative bacillus meningitis are most commonly related to nosocomial infections associated with head trauma or neurosurgery, with community-acquired gram-negative bacillus meningitis being quite infrequent [3]. Herein, we report on the clinical manifestations, laboratory data, and outcomes of therapy of a 66-year-old man with spontaneous community-acquired *Proteus mirabilis* meningitis.

## Case presentation

A 66-year-old man with a past medical history of the neurogenic bladder due to remote spinal cord trauma, recurrent urinary tract infection (UTI), chronic lower back pain on regular caudal epidural steroid injections, and status post spinal cord stimulation (SCS) implant five years prior to the admission with device failure presented to the emergency department reporting a two-day history of severe headache, fever, and confusion. The patient also endorsed vomiting and mild right upper quadrant (RUQ) pain. The patient reported worsening of chronic back pain, described as shooting, 8 out of 10, with radiation to both thighs. Of note, the patient had been diagnosed with a *P. mirabilis* UTI a week prior to the current presentation and was appropriately treated with five days of cephalexin. Vital signs revealed the following: temperature, 101.9 °F; pulse, 95 bpm; blood pressure, 184/73 mmHg; and SpO2, 95% in room air. The patient was alert and oriented, and pupils were symmetric and reactive. The neck was supple, without rigidity. Cardiac and pulmonary exams were unremarkable. The abdominal examination revealed mild tenderness on the RUQ, and Murphy’s sign was negative.

Laboratory tests on the day of admission demonstrated a blood leucocyte count of 11.4 × 109/L and a C-reactive protein of 39.7 mg/dL. The creatinine level was 0.69 mg/dL, the lactic acid level was 1.5 mmol/L, liver function tests were within normal limits, and the blood sodium level was 140 mmol/L. Cerebrospinal fluid (CSF) was described as yellow and xanthochromic, which has pleocytosis of 1,637 cells/uL with a differential of 98% polymorphonuclear cells (PMN) and 2% mononuclear cells (Table 1). The CSF glucose level was 3 mg/dL, and the total protein level was 958 mg/dL. No organism was seen on the Gram stain. CSF culture revealed few pan-susceptible *P. mirabilis*. Urinalysis was normal. Brain computed tomography (CT) was normal, except for mild mucosal thickening of the maxillary sinuses, sphenoid sinuses, multiple ethmoid air cells, and left frontal sinus, compatible with mild sinus disease. CT abdomen with contrast and US abdomen were unremarkable except for cholelithiasis with minimal gallbladder and common bile duct distention. A hepatobiliary nuclear medicine scan (HIDA) showed late filling of the gallbladder compatible with chronic cholecystitis.

**Table 1:** Cerebral spinal fluid analysis D0, admission day; D7, seventh day on antibiotic therapy; D30, readmission day; RBC, red blood cell

	Latest reference range & units	D0	D7	D30
Glucose	50-75 mg/dL	3	10	12
Protein, total	15-45 mg/dL	958	604	279
Color	Colorless	Yellow	Yellow	Colorless
Character	Clear	Slightly hazy	Hazy	Hazy
Volume	mL	2	15	7
Nucleated cells	0-5/uL	1,637	3,284	3,985
RBC	0/uL	1,000	3,000	<1,000
Polymorphonuclear	%	98	93	91
Mononuclear	%	2	2	7
Gram	Negative	Negative	Negative	Negative
Bacterial culture	Negative	Proteus mirabilis	Negative	Negative

The patient was initially started on empiric antimicrobial therapy with acyclovir, ampicillin, ceftriaxone, vancomycin, and metronidazole. After the isolation of *P. mirabilis* on CSF culture, the anti-microbial treatment was narrowed down to ceftriaxone 2g twice daily.

The patient defervesced after initiation of antibiotics. By hospital day 1, confusion, headache, and vomiting had improved. However, the patient complained of worsening of his chronic lower back pain. Follow-up CSF analysis on day 7 of antibiotic therapy showed a persistent pleocytosis, high protein level, and low glucose; however, the CSF culture did not reveal any bacteria (Table 1). We suspected that the SCS device might have represented the route of entry of a genitourinary pathogen into the CSF. As the device was not MRI-safe and the patient had not used it, the provider team decided to explant it. Lumbar spine MRI did not reveal any sign of osteomyelitis, epidural abscess, or any impending neurological condition. Culture swabs from the SCS pocket and from the device itself were negative. Back pain improved after adjustment of pain medication. The patient was discharged to complete a 21-day regimen of ceftriaxone at home.

Nine days after finishing his antibiotic regimen, however, our patient was readmitted to the hospital due to recurrence of headache and fever (100.7 °F) associated with neck rigidity. Repeat CSF studies (Table 1) revealed persistent pleocytosis with 3,985 nucleated cells (91% PMN); protein level, 279 mg/dL; and glucose level, 12 mg/dL. Gram stain and CSF culture were negative, as well as urine and blood culture. The patient was restarted on ceftriaxone 2g twice a day. The patient had significant improvement in fever, headache, and neck rigidity two days after resuming antibiotic therapy. He was discharged home to complete an additional 42-day regimen of ceftriaxone. One month after finishing the antibiotic regimen, the patient was seen and remained afebrile with no sign of recurrent meningitis symptoms.

## Discussion

The causative pathogens of meningitis differ among different patient populations. Community-acquired infections are frequently caused by *S. pneumoniae*, *H. influenzae*, *L. monocytogenes*, *N. meningitidis*, and group B streptococci. Since the advent of childhood vaccination programs, a decrease in incidence and mortality associated with meningitis has been noticed in high-income countries. Worldwide, *S. pneumoniae* and *N. meningitidis* are the most common pathogens in adults [4]. Non-*H. influenzae* gram-negative rods rarely cause spontaneous meningitis in adults. Our patient was found to have spontaneous community-acquired meningitis caused by *P. mirabilis*.

Most cases of gram-negative bacillary meningitis occur in neonates and infants [2]. Reports of meningitis in adults caused by gram-negative bacillus have been limited. Two patterns have been noticed: the first pattern is seen post-neurosurgical/head trauma, and the second one is a spontaneous form in elderly patients with comorbidities such as diabetes, cirrhosis or malignancy, and UTIs. Berk and McCabe published a series of 30 adults with meningitis caused by gram-negative bacilli admitted to four hospitals in 1968 and 1978 in the US [5]. *Escherichia coli* was the most frequent etiologic agent, followed by *Klebsiella pneumoniae*, *Acinetobacter calcoaceticus*, *H. influenzae*, and *Pseudomonas aeruginosa*. *P. mirabilis* was isolated from only one patient with meningitis [5].

In neonates, it is believed that these infections occur by bacterial hematogenous spread of the organism to the brain or meninges. Nevertheless, contiguous spread to the brain from localized infections has also been reported [6]. Our patient was diagnosed with UTI caused by the same bacterial agent seen in his CSF seven days before symptoms of his central nervous system (CNS) infection began. Therefore, in our case, we hypothesize that the urinary tract was the original source of the infection that spread hematogenously or contiguously to the meninges. This is compatible with what was seen in previous studies. Berk and McCabe found that the urinary tract appeared to be the original focus of infection in seven of the 15 patients with spontaneous meningitis caused by gram-negative bacteria, with the urine culture revealing the same organism found in the CSF [5]. Pomar et al. also associated instrumentation of the urinary tract and UTI with portals of entry in cases of spontaneous gram-negative meningitis [7].

Another well-known potential mechanism of meningeal infection is the direct inoculation of a pathologic agent during neurologic procedures, leading to an epidural abscess or lumbar discitis followed by meningeal dissemination [8]. Our patient had frequent epidural injections as part of the treatment for chronic back pain. However, there was no evidence of spinal or epidural infection on his MRI exam.

We also considered whether the presence of a tunneled spinal cord stimulator in close contact with the epidural area could have been related to our patient’s atypical meningeal infection by *P. mirabilis*. Infections of SCS devices have been reported, but they usually reflect inoculation of the implant or generator pocket with microorganisms from the patient’s skin flora or contaminated aerosol. In most studies, the time from device implantation to SCS infection was <90 days. This argues against that hypothesis in our patient because his SCS device had been implanted more than five years prior to his meningeal infection. As far as we know, only a single case of meningitis related to SCS implant has been reported [9]. Due to the anatomical location of SCS devices and the fact that none of the device components are endovascular, hematogenous seeding of these devices from bacteremia arising from a non-SCS site is highly unlikely [10].

Once *P. mirabilis* is identified on CSF culture, the antibiotic treatment should be directed according to susceptibility testing and the agents used should have good CNS penetration [11]. Most *Proteus* CNS infections are susceptible to third-generation cephalosporins, but cases with extended spectrum beta-lactamase have been reported, which were treated with carbapenems [12].

There is a lack of high-level evidence recommendations regarding the optimal duration of antibiotic therapy in this scenario. Based only on observational studies, the current Infectious Diseases Society of America’s Clinical Practice Guidelines recommends that gram-negative bacillary meningitis should be treated for 10-14 days (strong recommendation and low level of confidence), but some experts suggest treatment for 21 days (weak recommendation and low level of confidence). It is also recommended to repeat CSF analysis for five to seven days after beginning antibiotics. The treatment should be continued for 10-14 after the last positive culture (strong recommendation and low level of confidence) [11]. Our patient did have a repeat lumbar puncture on hospital day 7 that revealed significant CSF pleocytosis and hypoglycorrhachia, but with no bacterial growth in the culture. Despite 21 days of ceftriaxone and a sterile control CSF, our patient did relapse and was readmitted with meningitis symptoms. Our patient required an additional 42 days of ceftriaxone to finally achieve sustained symptom remission.

## Conclusions

Spontaneous community-acquired *P. mirabilis* meningitis is rare among adult patients. However, patients with recurrent UTI or urinary tract instrumentation may have a higher risk of developing this condition. Early diagnosis and treatment are pivotal to prevent complications and disease mortality. Even after completion of antibiotics, these patients should be followed clinically because the rate of relapse is high. Sterilization of the CSF and extended antibiotic therapy are crucial to prevent recurrences.
